# Interaction between the land and the sea: sources and patterns of nutrients in the scattered coastal zone of a eutrophied sea

**DOI:** 10.1007/s10661-018-7143-z

**Published:** 2018-12-19

**Authors:** M. Raateoja, P. Kauppila

**Affiliations:** Marine Research Centre, Finnish Environment Institute, Latokartanonkaari 11, 00790 Helsinki, Finland

**Keywords:** Baltic Sea, Nutrient, Eutrophication, Long-term, Trend

## Abstract

A long-term trophic development of three geographical transects—including a river mouth, an estuary, and an archipelago—were studied in the southern Finnish coast in the Baltic Sea. Each transect was studied to clarify how far off the coast the land-based nutrient sources (catchment factor, CF) had a decisive role in shaping the wintertime regimes of dissolved inorganic nitrogen and dissolved inorganic phosphorus and where the marine processes (marine factor, MF) start to play a major role. Generally, CF controlled the nutrient regime from the coast to the outer brink of the inner coastal area, after which MF started to dominate. The estuaries exhibited steep vertical nutrient gradients, above which the riverine input dominated the nutrient regime. The extent of the area where CF dominated the nutrient regime was therefore decisively dependent on estuarine stratification, i.e., whether the conclusions were drawn based on the surface layer data, including the riverine impact, or on the data beneath that layer, including the marine impact. This result deviates from the current consensus that the trophic regime of the sea is most directly assessed by the surface layer nutrient content. The estuarine nutrient regime is unrepresentative to that of a typical coastal water body due to the strong land-based impact on the estuary. Therefore, any generalization of the trophic condition of an estuary to represent areas farther off the coast should be done cautiously. The estuaries should also be defined as belonging to transitional waters according to the typology related to European Marine Legislation.

## Introduction

The Baltic Sea (BS) is one of the most eutrophied seas worldwide (HELCOM 2014, 2009) and is subject to marine management stipulated by the EU Marine Strategy Framework Directive (EU MSFD, EU 2008) and coordinated by Baltic Marine Environment Protection Commission (HELCOM). The MSFD also covers coastal waters where the implementation and management follows the EU Water Framework Directive (EU WFD, EU 2000). As part of the process, the trophic state of the BS has been assessed on a regular basis in the scale of the sea (e.g., HELCOM 2017) or a sub-basin (e.g., Raateoja and Setälä 2016). This is the proper scale for resolving how to mitigate the eutrophication of the BS in the path towards its good environmental state and sustainable use (HELCOM 2007). The basin-scale management ignores the local coastal scale where the land-based nutrient sources and the coastal-offshore interaction have often a more profound impact on the trophic development than have the marine processes.

The nutrient regime along the coastal-offshore gradient tends to behave conservatively (Officer 1979; Officer and Lynch 1981), changing gradually from the coastal condition to the offshore one. This is caused by dilution and the “filter effect” where a part of the land-based nutrient stock is transformed and/or retained in the coastal zone mainly by denitrification, permanent burial, and plant assimilation (Asmala et al. 2017; Hellemann et al. 2017). This effect has been quantified both for tidal oceanic estuaries (Nixon et al. 1996; Jickells et al. 2014) and virtually non-tidal brackish water estuaries like in the Baltic Sea (Almroth-Rosell et al. 2016; Asmala et al. 2017). The spatial extension of the land-based impact on the nutrient regime depends on the openness of the coast. In an open coastal area, this impact does not typically extend to far off the coast because of the weak “filter effect” and effective dilution. Riverine or point-source nutrient flow is quickly damped there by the large-scale marine current patterns. In a scattered coastal area, in turn, this impact can extend much farther off the coast and the nutrient regime of a sheltered archipelago can differ drastically from that in the offshore. The alongshore cyclonic currents of offshore origin do not have much impact there, and hence, the water exchange with the offshore is at times severely restricted. The “filter effect” has a larger time window to operate there too.

Within the BS, large archipelago areas can only be found along parts of Swedish and Finnish coasts (Asmala et al. 2017). There, the character of granite rock formations (Winterhalter et al. 1981) creates a great number of islands and islets but affects also coastal topography to produce an extremely irregular coastline and a myriad of small estuaries. Poor lateral water exchange together with temperature-driven water stratification has led to oxygen deprivation in the local deeps in the southern Finnish archipelago (Conley et al. 2011; Wallius 2006).

The discussion of the roles of the catchment and the sea as sources for nutrients in a scattered coastal area underlines the fundamental role of estuarine processes. Estuaries are environments where the anthropogenic impact by land drainage on the marine system is at its strongest (Pritchard 1967). Strict attention has been drawn to the environmental state of the estuaries as long as the coastal areas have been consistently monitored. The contemporary socioeconomic climate emphasizes cost-effectiveness in the environmental front, including monitoring (Nygård et al. 2016). The monitoring network should offer a good return value for the resources spent on monitoring by being able to reliably describe the characteristics of the monitored area. This is a question of sampling frequency but also a choice of sampling locations. Estuaries have special hydrodynamic and/or topographic characteristics (Pritchard 1967) and are often overwhelmingly impacted by the land-based nutrient load as compared to coastal areas in general, questioning the representativeness of the estuarine datasets for the management of coastal seas.

## Material and methods

Our dataset covered three geographical transects possessing a coastal-offshore gradient in the southern Finnish coast of the BS. They all have been subject to consistent long-term monitoring efforts. Using this unique dataset in terms of both in its length in time and sampling frequency, we wanted to find out (i) how far off the coast the trophic development was predominantly dictated by the processes taken place in the catchment and where the marine processes started to play a major role and how the characteristics of an estuary affected the outcome and (ii) how well the estuarine nutrient data (wintertime inorganic phosphorus and nitrogen) described the trophic development of the surrounding coastal area.

### Study area

**Fig. 1:**
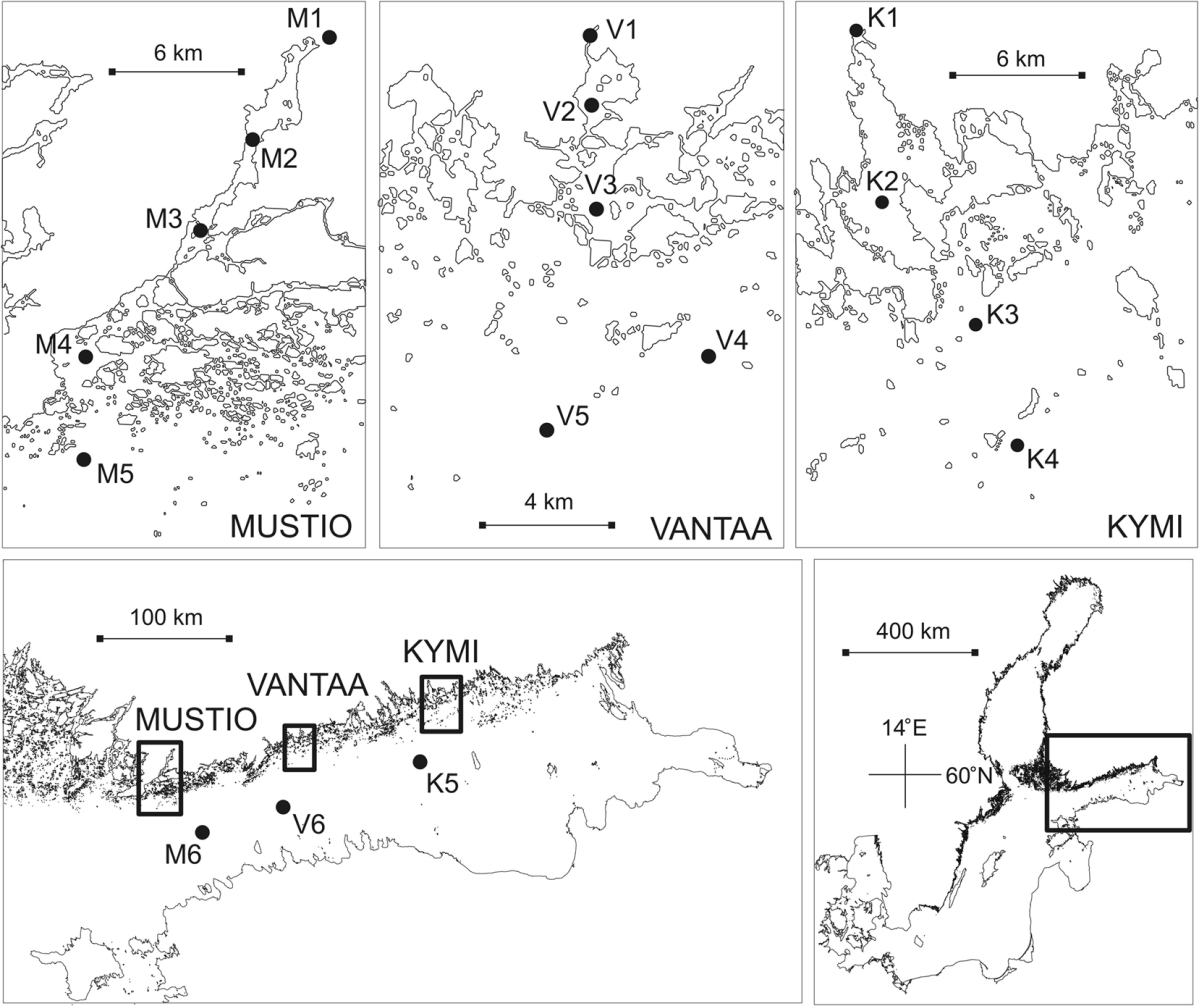
The study area and the cases MUSTIO, VANTAA, and KYMI. The stations were named according to the regional initials (MUSTIO = M, VANTAA = V, and KYMI = K) and numbers according to the distance from the river mouth

The cases of this study consist of a transect covering a river mouth, an estuary with its adjacent archipelago, and the offshore Gulf of Finland waters in the BS (GOF; Table 1 and Fig. 1). The cases are named according to the main discharging rivers as follows: MUSTIO (the River Mustionjoki), VANTAA (the River Vantaanjoki), and KYMI (the River Kymijoki). We denoted the stations according to the regional initials (MUSTIO = M, VANTAA = V, and KYMI = K) and numbers according to the distance from the river mouth (Table 2).

**Table 1:** The study cases

	MUSTIO	VANTAA	KYMI
Catchment area (km^2^)	2046	1686	37158
Lake area (%)	12.2	2.3	18.3
Agricultural area (%)	20.8	24.3	8.8
Catchment retention (%)^a^	66 TOTP, 52 TOTN	6 TOTP, 10 TOTN	74 TOTP, 54 TOTN
Main lake basins	Lake Lohjanjärvi (88, 12.7)	Lake Tuusulanjärvi (5.9, 3.2)	Lake Päijänne (1118, 16.2)
	Lake Hiidenvesi (30, 7.2)	Lake Kytäjärvi (2.7, 4.5)	Lake Keitele (493, 6.8)
Main river	River Mustionjoki	River Vantaanjoki	River Kymijoki
Average flow (m^3^ s^−1^)	17	16	313^b^ (158^c^)
TOTN load (t year^−1^)	525	1278	6372^b^ (3470^c^)
TOTP load (t year^−1^)	19	62	197^b^ (120^c^)

Parentheses below the lakes: surface area (km^2^), mean depth (m). Riverine data: data bank of the Finnish Environment Institute

^a^Huttunen et al. (2016)

^b^The River Kymijoki in its entirety

^c^Only the westernmost outlet that discharges to the Gulf of Finland via Ahvenkoski Rapids

**Table 2:** The stations in the study

	MUSTIO	VANTAA	KYMI
River	M1	V1	K1
	Mustionjoki 4,9	Vantaa 4,2	Ahvenkoski 001
	0–1 m, 0.0, 0.0	0–1 m, 0.0, 0.0	0–1 m, 0.0, 0.0
Inner archipelago	M2	V2	K2
	UUS-16	Vanhankaupunginselkä 4	Kyvy-9
	41 m, 1.5, 4.9	2 m, 1.6, 2.3	16 m, 1.1, 4.5
	M3	V3	
	Stadsfjärden 98 +	Vasikkasaari 18	
	Stadsfjärden Vitsten 210	19 m, 4.8, 5.6	
	6 m, 2.7, 5.0		
Outer archipelago	M4	V4	K3
	Storfjärden	UUS-10A	Kyvy-1
	35 m, 5.5, 6.4	53 m, 5.3, 6.3	28 m, 3.5, 5.5
	M5	V5	K4
	UUS-23	39A	Kyvy-10
	60 m, 5.9, 6.9	41 m, 5.3, 6.5	40 m, 4.2, 5.7
Offshore	M6	V6	K5
	LL9	LL7	LL3A
	69 m, 5.7, 8.8	100 m, 6.5, 10.2	68 m, 4.8, 8.2

Upper row: station abbreviation. Middle row: the official station name. Lower row: station depth (m), salinity at the surface (g kg^−1^), salinity near to the seafloor (g kg^−1^)

MUSTIO has a moderate riverine impact, a large estuary, and an extensive archipelago (Table 1). The River Mustionjoki (M1; Table 2) discharges into the estuary, the Pojo Bay, which is a semi-enclosed inland bay. It is by definition a fjord-type estuary (sensu Pritchard 1967) where riverine input exceeds episodic marine inflow; fresher water floats seaward on top of the more saline water layer. The deep area of the Bay (M2) has a strong halocline, because the deep water mass receives episodic inflows of more saline water (Malve et al. 2000; Stipa 1999). The residence time of the Bay is quite long (1.5 years; Meeuwig et al. 2000), and the deep water layer experiences intermittent stagnation periods especially in the summertime, leading to hypoxic conditions (Stipa 1999). The halocline extends to the shallow inner coastal area (M3). The water gradually deepens when entering the outer archipelago (M4). M5 represents the outmost reaches of the coastal area, and M6 is an offshore station.

VANTAA has a moderate riverine impact, a small estuary, and a restricted archipelago (Table 1). Above all other regions here, it has been subject to a pronounced anthropogenic impact (Lappalainen and Pesonen 2000; Varmo et al. 1989). The River Vantaanjoki (V1; Table 2) discharges to the semi-enclosed and turbid (Secchi depth < 0.5 m) estuary, the Vanhankaupunginlahti Bay (V2). It is oligohaline due to a restricted water exchange with the GOF and weakly stratified due to shallow waters making it subject to efficient wind-induced mixing. Regardless of the heavy load history, the benthic release of nutrients does not occur in the Bay because of its short residence time (3 months; Meeuwig et al. 2000). The waters flow through the narrow strait to the inner archipelago (V3) that is subject to a strong marine impact (salinity ≈ 5 g kg^−1^) but also to a strong riverine impact especially during strong riverine flow events. Further off the coast, V4 and V5 are located in the outer archipelago. V6 is an offshore station.

KYMI has a pronounced riverine impact, a large estuary, and a restricted archipelago. It has a large catchment including large lakes and a pronounced riverine input (Table 1). The westernmost outlet of the River Kymijoki (K1; Table 2) discharges into the estuary, the Ahvenkoskenlahti Bay (K2), with an average water flow of ten times the other two flows. The Bay is by definition a salt wedge estuary (sensu Schijf and Schönfeld 1953) where riverine inflow exceeds marine inflow. It has ridge formations at its entrance that, however, cannot restrict lateral water exchange below the halocline locating at the depth of 3–5 m (Pitkänen 1994; Pitkänen et al. 1986). Lateral and vertical mixing processes, leading to the residence time of only 1 week, prevent any oxygen deprivation occurring in the seafloor (Pitkänen et al. 1986; Meeuwig et al. 2000). K3 is situated at the outer coastal area and K4 farther at the outer brink of the archipelago. The riverine impact on this area is strong (Pitkänen 1994), and based on the observed salinity level, K3 could be chemically classified as an inner archipelagic station. K5 locates in the offshore area.

### The dataset

The field sampling and subsequent laboratory analyses for the riverine and coastal datasets were carried out, or in the case of statutory monitoring, coordinated by (i) the South-East Finland Centre for Economic Development, Transport and the Environment, (ii) Uusimaa Centre for Economic Development, Transport and the Environment, (iii) City of Helsinki Environment Centre, (iv) Kymijoen vesi - ja ympäristö ry, (v) Länsi-Uudenmaan vesi ja ympäristö ry, and (vi) Tvärminne Zoological Station of Helsinki University, as well as by their organizational predecessors. The offshore dataset was collected and analyzed by the Finnish Institute of Marine Research and the Finnish Environment Institute as part of the HELCOM coordinated monitoring program.

The dissolved inorganic nitrogen (DIN) and dissolved inorganic phosphorus (DIP) were analyzed colorimetrically (Grasshoff et al. 1999). DIN consisted of nitrate and nitrite sum [NO_2_ + NO_3_] and ammonium (NH_4_), i.e., DIN = [NO_2_ + NO_3_] + NH_4_. DIP equaled phosphate (PO_4_). The determination of NO_2_ + NO_3_ and PO_4_ has changed from a manual determination to the flow infection analysis (FIA) or the continuous flow analysis (CFA) in the late 1980s to the early 2000s, depending on a laboratory, while NH_4_ is still routinely determined manually in these laboratories.

Salinity was measured from discrete water samples with various Autosal bench salinometers calibrated against IAPSO standard seawater (Grasshoff et al. 1999). The use of various CTD systems became more popular towards to the late stages of the study. The CTD-based salinity was measured in situ with a conductivity cell.

DIN and DIP data were collected at the upper 10 m (riverine stations had only 0–1 m monitoring depth) in December–March. The molar DIN/DIP ratio was calculated using these data. Whenever temporal dimension was employed, the data was compiled based not on calendar years but winters. Thus, the samples taken in, e.g., December 1981 were included in the dataset of the winter 1981–1982 (simply 1982).

Station-wise, observations outside the region of average ± two times standard deviation were excluded from the dataset in order to prevent riverine flood peaks (DIN) and strong estuarine mixing events (DIP) from contributing to the conclusions.

The cases included estuaries where the river plume dominates the chemical contents of the surface water especially in the wintertime. For instance, the influence of snow melt waters in MUSTIO can be detected as far as at M4 (Heiskanen and Tallberg 1999; Niemi 1975), and a river plume in KYMI can be up to 3 m thick at K3 during the snow melt (Pitkänen et al. 1990). Therefore, the station data were visually checked whether there was any notable discrepancy in the nutrient regimes between the surface layer and the layer beneath, and sampling depths were excluded from the final dataset whenever needed.

All the estuaries and their adjacent archipelagos in this study freeze every winter. This extends the geographical impact of river plumes on this study that is based on wintertime monitoring data. Sampling during high or low tide, in turn, is not an issue in a virtually non-tidal BS (Leppäranta and Myrberg 2009).

### The impact of analytical changes

We tested whether the variation produced by the technical changes in the analytical procedures had any impact on the long-term datasets. We collected the metadata of the stations including the detailed notes of sample pre-treatment and analytics and pin-pointed the times of technical changes in the analytical procedure. We chose two stations from each region and calculated the annual averages for DIN and DIP and further their differences between two consecutive years (excluding the times of analytical changes). For DIN, the testing parameter $\overline{a_{\mathrm{year}}}$ (μmol L^−1^)—giving notion of the natural DIN variation between the years—was defined as:

1 $$ \overline{a_{\mathrm{year}}}=\left({\sum}_{i=1}^n\left|{\mathrm{DIN}}_{i+1}\hbox{--} {\mathrm{DIN}}_i\right|\right)/n $$

where *i* is the starting year of monitoring.

DIN (excluding NH_4_) had two technical changes in the analytics, both of them being related to shifts from manual to automated determination. The parameter $\overline{a_{\mathrm{change}}}$ (μmol L^−1^) for DIN was the average of the DIN differences between the year before and the year after the technical change in the analytics and was defined as:

2 $$ \overline{a_{\mathrm{change}}}=\left(|{\mathrm{DIN}}_{x+1}-{\mathrm{DIN}}_{x-1}|+|{\mathrm{DIN}}_{y+1}-{\mathrm{DIN}}_{y-1}|\right)/2 $$

where *x* is the year of the first technical change in the analytics and *y* is the year of the second one. $\overline{a_{\mathrm{change}}}$ gives a notion of the DIN variation due to the combined effect of technical changes in the analytics and the nature.

DIP was handled as DIN except that DIP had only one technical change in the analytics, being also related to a shift from manual to automated determination. So, for DIP, $\overline{a_{\mathrm{change}}}$ was defined as:

3 $$ \overline{a_{\mathrm{change}}}=\left|{\mathrm{DIP}}_{x+1}\hbox{--} {\mathrm{DIP}}_{x-1}\right| $$

where *x* is the year of the technical change in the analytics. The reasoning whether the analytical changes produced a variation to be taken into closer examination was as follows:

4 $$ \Big\{{\displaystyle \begin{array}{c}\overline{a_{\mathrm{change}}}<\left(\overline{a_{\mathrm{year}}}+{\mathrm{SD}}_{\mathrm{year}}\right)\Rightarrow \mathrm{no}\ \mathrm{attention}\ \mathrm{needed}\\ {}\overline{a_{\mathrm{change}}}>\left(\overline{a_{\mathrm{year}}}+{\mathrm{SD}}_{\mathrm{year}}\right)\Rightarrow \mathrm{no}\ \mathrm{attention}\ \mathrm{needed}\end{array}} $$

SD_year_ is the standard deviation of a time series consisting on the differences of DIN or DIP between two consecutive years.

### Land-based and marine impact on the stations

The dataset was based on station visits and hence allowed us to estimate not the actual point but rather the boundary area between two points in a coastal-offshore gradient where the sea overrode the catchment as the main contributing factor shaping the nutrient regime. The processes taken place in the catchment are referred henceforth as the catchment factor (CF) and the marine processes as the marine factor (MF).

There were two diagnostics for determining this boundary area. First, the variation of a molar DIN/DIP ratio in the coastal-offshore gradient was used, as opposed to either the riverine or the offshore station. Second, a linear regression analysis was used on the temporal DIN or DIP datasets of the adjacent stations. Only those years were selected for the analysis from which there were observations from both of the adjacent stations. A *p* value < 0.05 in a linear regression fit was considered as a criterion for a statistically significant deviation from the null hypothesis H_0_: *β* = 0. Here, *β* is a slope coefficient of the linear fit for the temporal datasets between the two neighboring stations. For each station, standardized values were used in the regression analysis as follows:

5 $$ {\displaystyle \begin{array}{l}\mathrm{standardized}\ \mathrm{value}=\\ {}\left(\mathrm{original}\ \mathrm{value}-\mathrm{average}\ \mathrm{of}\ \mathrm{the}\ \mathrm{time}\ \mathrm{series}\ \mathrm{data}\right)\times {\left(\mathrm{SD}\ \mathrm{of}\ \mathrm{the}\ \mathrm{time}\ \mathrm{series}\ \mathrm{data}\right)}^{-1}\end{array}} $$

where SD refers to the standard deviation.

## Results

### Impact of river plumes

**Fig. 2:**
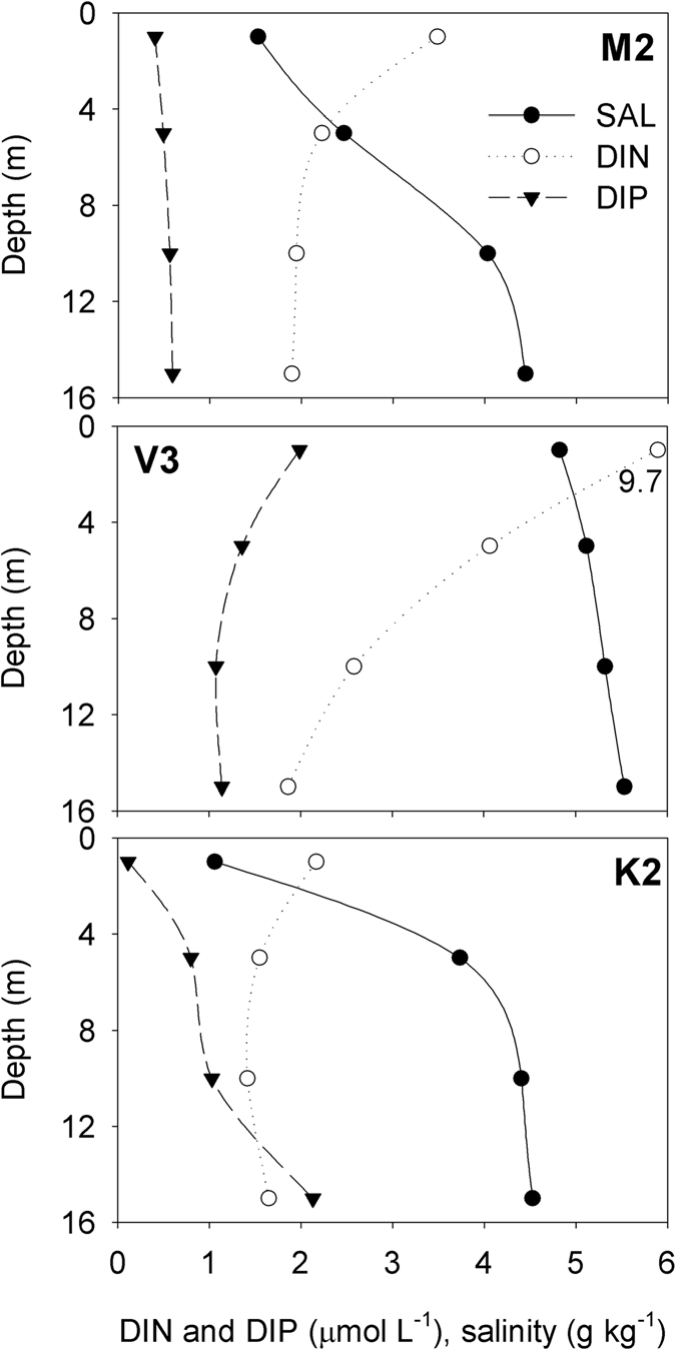
Salinity, DIN, and DIP as a function of depth in M2, V3, and K2 as examples of the riverine effect. DIN values were divided by ten for presentation purposes

The surface (0 or 1 m) measurements of DIN and DIP at the stations M2, M3, M4, V3, K2, and K3 deviated clearly from the general hydrochemical setup (Fig. 2). Based on the visual interpretation of vertical nutrient patterns, the surface layer measurements (0 and/or 1 m) were excluded from the final dataset at these stations. These measurements were examined separately to clarify the impact of the river plume. The use of surface layer data instead of the original data never shrank the geographical predominance of the catchment processes in the coastal nutrient regime and clearly stretched it seaward whenever a river plume was considerable.

### Impact of analytical changes

The technical changes in the analytical procedures of DIN and DIP took place in the late 1980s to the early 2000s. The environmental testing laboratories contributing to the dataset acted largely in concert when employing new technical and/or chemical developments for the procedures.

We observed that $\overline{a_{\mathrm{change}}}$ > ($\overline{a_{\mathrm{year}}}$ + SD_year_) only for DIN at K1 and for DIP at V4 (Table 3). In these cases, the parameter value returned in the level observed before the analytical change in a couple of year time frame since the analytical change and most importantly clearly before the next analytical change occurred (data not shown).

**Table 3:** The impact of analytical changes on the representativeness of the dataset

	DIN (μmol L^−1^)				DIP (μmol L^−1^)			
	$\overline{a_{\mathrm{year}}}$	$\overline{a_{\mathrm{year}}}$ + SD_year_	Criterion	*a*_change_	$\overline{a_{\mathrm{year}}}$	$\overline{a_{\mathrm{year}}}\kern0.5em$+ SD_year_	Criterion	*a*_change_
K1	3.11	5.60	<	6.98	0.06	0.11	>	0.04
K3	1.33	2.31	>	0.35	0.13	0.24	>	0.04
V1	33.2	56.2	>	14.2	0.42	0.73	>	0.33
V4	1.92	3.78	>	1.97	0.15	0.26	<	0.29
M1	7.46	16.6	>	8.45	0.13	0.21	>	0.07
M5	1.44	2.60	>	0.43	0.12	0.53	>	0.08

The criterion for a case to be taken into further examination was ($\overline{a_{\mathrm{year}}}$ + SD_year_) < *a*_change_

Variation in the datasets stemming from the analytical changes, whether occurred in any measurable quantities, seemed to have been overridden by natural variation. Thus, we felt confident to ignore the role of analytical changes for the conclusions of this study.

### Trophic development and current state of the stations

For MUSTIO, we found a coastal-offshore gradient for DIN, DIP, and the DIN/DIP ratio (Table 4). The gradients were decreasing, increasing, and decreasing, respectively, with the distance from the coast. Similar gradients for DIN and the DIN/DIP ratio were found in VANTAA. The River Vantaanjoki in VANTAA carries substantial nutrient load relative to its flow, causing a decreasing DIP pattern with the distance from the coast. KYMI showed no clear patterns in its coastal-offshore gradient; here, the gradient appeared as an abrupt change in the DIP concentration level, and hence, in the DIN/DIP ratio between K1 and K2.

**Table 4:** DIN, DIP, and molar DIN/DIP ratio status since the year 2000 as averages ± SD

		MUSTIO	VANTAA	KYMI
DIN (μmol L^−1^)	1	44.7 ± 8.6	135.5 ± 38.1	24.2 ± 4.3
		*1972/167*	*1964/184*	*1984/201*
	2	27.1 ± 7.3	136.6 ± 59.9	15.0 ± 2.6
		*1968/103*	*1964/142*	*1983/48*
	3	14.5 ± 4.4	24.0 ± 12.6	12.5 ± 1.9
		*1996/20*	*1971/125*	*1979/122*
	4	6.6 ± 1.2	9.0 ± 1.9	12.5 ± 1.7
		*1996/196*	*1971/225*	*1987/19*
	5	6.8 ± 1.2	9.8 ± 1.0	10.2 ± 1.6
		*1978/159*	*1979/67*	*1975/106*
	6	8.0 ± 1.7	8.0 ± 1.8	
		*1975/99*	*1975/168*	
DIP (μmol L^−1^)	1	0.43 ± 0.14	1.38 ± 0.66	0.15 ± 0.09
		*1979/127*	*1974/167*	*1984 /128*
	2	0.47 ± 0.14	1.55 ± 1.28	1.08 ± 0.25
		*1975/160*	*1971/136*	*1983/18*
	3	0.81 ± 0.22	1.04 ± 0.20	1.05 ± 0.17
		*1996/22*	*1971/122*	*1979/115*
	4	0.82 ± 0.15	1.00 ± 0.20	0.86 ± 0.16
		*1996/200*	*1971/224*	*1987/20*
	5	0.85 ± 0.13	0.87 ± 0.20	0.90 ± 0.14
		*1978/163*	*1979/69*	*1975/109*
	6	0.75 ± 0.19	0.78 ± 0.17	
		*1975/105*	*1975/166*	
DIN/DIP ratio	1	103 ± 25	103 ± 28	187 ± 64
	2	55 ± 17	109 ± 48	13 ± 5
	3	19 ± 11	22 ± 6	13 ± 2
	4	9 ± 2	9 ± 2	15 ± 2
	5	8 ± 2	12 ± 3	11 ± 3
	6	11 ± 3	11 ± 2	

For K2 and k4, the data covers the 1990s. Numbers 1 to 6 refer to the stations in each region. The row below in italics: start year of the entire dataset/total number of observations to describe the representativeness of the dataset

**Fig. 3:**
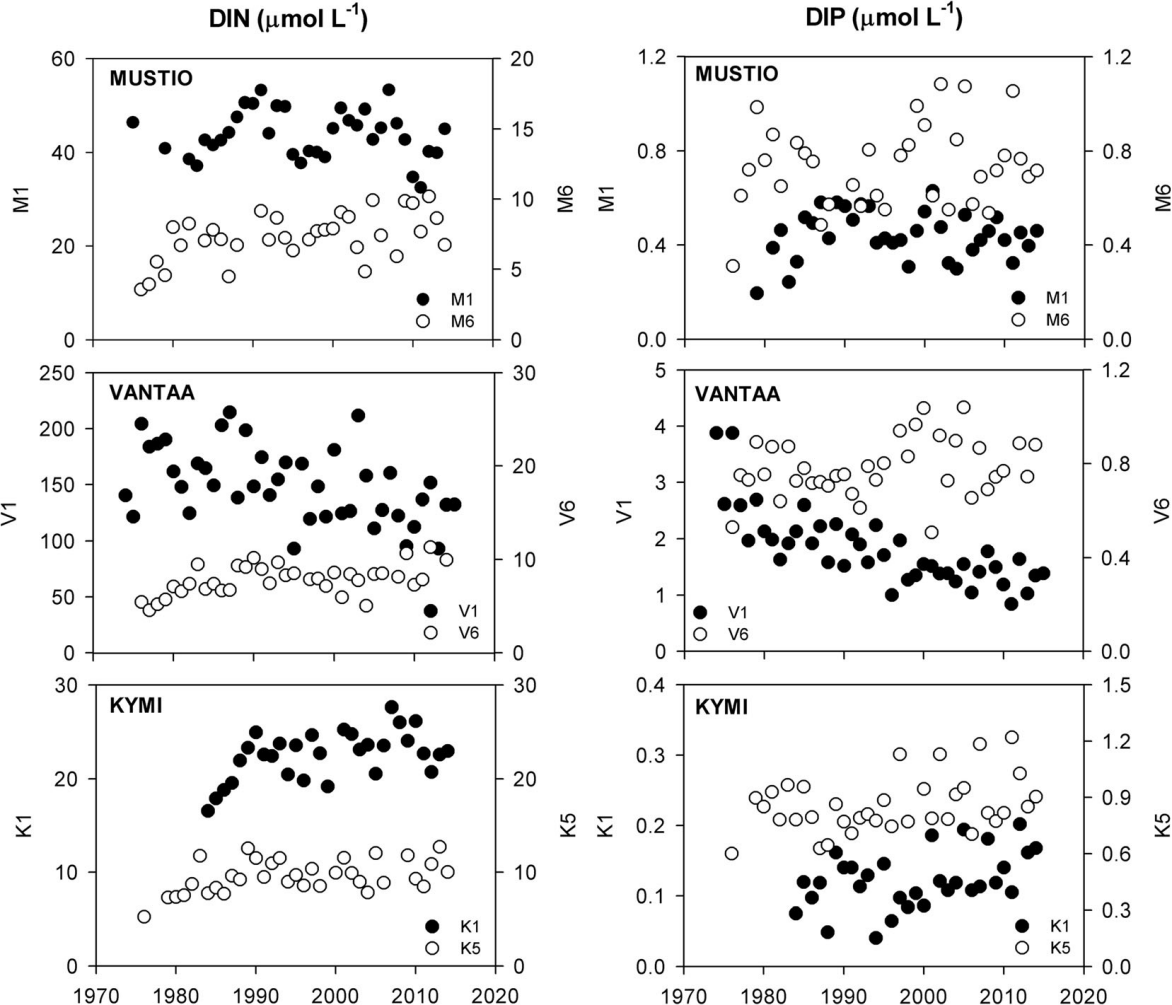
DIN and DIP trends in the riverine and offshore stations. Each dot represents the average wintertime condition (December–March). The stations were named according to the regional initials (MUSTIO = M, VANTAA = V, and KYMI = K) and numbers according to the distance from the river mouth

The offshore nutrient trends—represented by M6, V6, and K5—followed the general eutrophication development of the GOF (Fig. 3). The DIN concentrations increased in the 1970s and the early 1980s from the earlier levels of 4–6 μmol L^−1^, leveled out in the 1990s, and have stayed at an elevated level since then. The trends for the DIP concentration were less pronounced. The advancing eutrophication appeared as a higher inter-annual variation in DIP since the mid-1990s. On top of this temporal variation, the nutrient regime tended to increase eastwards from MUSTIO to KYMI. In the most recent situation, the wintertime surface DIN level ranged from 7–10 in the west to 9–11 μmol L^−1^ in the east (Table 4). For DIP, the corresponding levels fluctuated strongly, ranging from 0.7–1.0 in the west to 0.8–1.1 μmol L^−1^ in the east.

The nutrient trends of the rivers (represented by M1, V1, and K1) deviated much from each other. DIN and DIP concentrations in the River Mustionjoki have varied throughout the study period from 35 to 55 and 0.3 to 0.6 μmol L^−1^, respectively, exhibiting some alternating periods of higher and lower concentrations but without any distinct trends (Fig. 3). The River Vantaanjoki has showed steadily decreasing DIN and DIP concentration trends throughout the study period from almost 200 to somewhat over 100 and from 2.5 to 1 μmol L^−1^, respectively. DIN concentrations in the River Kymijoki increased in the 1980s and have not changed since then, ranging currently from 20 to 25 μmol L^−1^. DIP concentrations seem to have slightly increased throughout the study period from 0.10 to 0.20 μmol L^−1^, but the overall level was close to the limit of the analytical determination capability of the laboratories, especially towards the early phases of the study.

### Land-based and marine impacts on the stations

#### The River Mustionjoki

The location of the boundary area in MUSTIO differed depending on the parameter. The CF dominated landward from M3 according to DIN (*p* < 0.001 between M1 and M2 as well as between M2 and M3, i.e., for M1/M2/M3; Table 5). The MF, in turn, dominated the DIN pattern seaward from M4 (*p* < 0.05 for M4/M5/M6). Thus, the boundary area was located between M3 and M4 according to DIN. According to DIP, the MF dominated seaward from M3 (*p* < 0.001 for M3/M4/M5/M6). Thus, the boundary area was located between M2 and M3 according to DIP.

**Table 5:** Linear regression for the standardized estimates of annual DIN and DIP between the two adjacent stations as *r*^2^ (above) and *p* value *F*_1,*df*_ (below)

Station pairs	MUSTIO				VANTAA				KYMI			
	DIN	DIN_0–1 m_	DIP	DIP_0–1 m_	DIN	DIN_0–1 m_	DIP	DIP_0–1 m_	DIN	DIN_0–1 m_	DIP	DIP_0–1 m_
1 <> 2	**0.30**	**0.50**	0.01	**0.33**	**0.20**	**0.15**	**0.33**	**0.35**	0.01	**0.38**	0.04	0.07
	12.3_1, 29_***	36.7_1, 37_***	0.4_1, 29_	14.7_1, 30_***	8.0_1, 33_**	5.9_1, 35_**	15.4_1, 31_***	16.7_1, 31_***	0.3_1, 22_	13.8_1, 23_***	0.3_1, 9_	1.5_1, 20_
2 <> 3	**0.51**	**0.65**	0.00	**0.68**	**0.42**	0.08	**0.69**	**0.38**	0.04	**0.26**	*0.38*	0.00
	15.7_1, 15_***	56.6_1, 31_***	0.1_1, 16_	38.2_1, 18_***	23.1_1, 32_***	3.0_1, 33_	66.3_1, 30_***	17.8_1, 29_***	0.8_1, 18_	5.8_1, 17_*	6.2_1, 10_*	0.0_1, 19_
3 <> 4	0.04		*0.56*		0.01		0.00					
	0.6_1, 16_		19.1_1, 15_***		0.5_1, 32_		0.0_1, 31_					
4 <> 5	*0.36*		*0.73*		*0.40*		*0.53*					
	7.3_1, 13_**		37.0_1, 14_***		11.1_1, 17_**		22.9_1, 20_***					
5 <> 6	*0.14*		*0.38*		*0.34*		*0.66*					
	3.2_1, 19_*		12.1_1, 20_***		10.7_1, 21_**		40.8_1, 21_***					

Also included the same variables for 0–1 m data in the inner coastal area. Those zones are bolded where the CF appeared to be dominant, and those are italicized where the MF did the same. The station K4 was excluded from this analysis due to too scarce data, and hence, Kymi had only the inner coastal area included

*< 0.05, **< 0.01, ***< 0.001

**Fig. 4:**
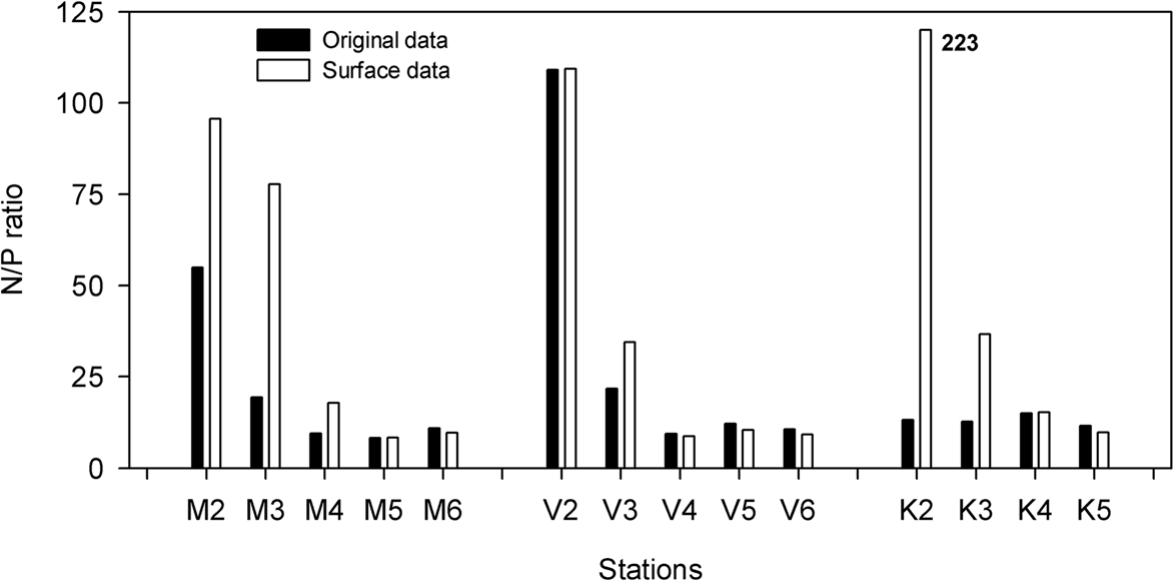
The average DIN/DIP ratio at the stations based on either the original data (0 and 1 m samples excluded) and the surface layer data (only 0 and/or 1 m data). The dataset, from which the average ratio was calculated, included annual representative values since the year 2000, except for K2 and K4 the data covers the 1990s. Depending on the station, the number of observations in the dataset varied from 8 to 16, and the coefficient of variation ranged from 20 to 30%. This comparison was not possible for the riverine stations. The stations were named according to the regional initials (MUSTIO = M, VANTAA = V, and KYMI = K) and numbers according to the distance from the river mouth

The DIN/DIP ratio > 50 at M2 and an elevated ratio at M3 compared to the offshore level indicated the impact of the CF at those stations, while the ratio settled to the offshore levels at M4, corroborating the dominant MF seaward from M4 (Table 4 and Fig. 4). No marked linkage was found for DIP between M2 and M3, suggesting that the deep water inflow into the estuary, the Pojo Bay, seems to be an episodic and/or only a moderate source of DIP.

The change of data source to the surface layer data increased the DIN/DIP ratio at M2 and M3 to be two to four times the ratio based on the original data, suggesting for a presence of waters high in N and poor in P there (Fig. 4). In the original approach, there were no predominance of the CF according to DIP landward from M3, but the impact became dominant in the surface layer data (*p* < 0.001 for DIP for M1/M2/M3; Table 5).

Waters in the surface layer at M2 and M3 were markedly of freshwater origin. As the original data provided two boundary areas—between M2 and M3 or between M3 and M4—the use of the surface data suggested explicitly the area to locate between M3 and M4.

#### The River Vantaanjoki

The boundary area in VANTAA was the most straightforward to determine. The CF dominated landward from V3 according to both DIN and DIP (*p* < 0.01 for V1/V2/V3; Table 5). There were no linkages between V3 and V4 either for DIN or DIP, indicating that the domination of the CF ended and the domination of the MF started somewhere between V3 and V4. The MF dominated seaward from V4 according to both DIN and DIP (*p* < 0.01 for V4/V5/V6). The DIN/DIP ratio of > 100 at V2 and an elevated ratio at V3 compared to the offshore level indicated an impact of the CF at those stations, while the ratio settled to the offshore levels at V4, corroborating the dominant MF seawards from V4 (Table 4 and Fig. 4).

When surface layer data was used, the DIN/DIP ratios at V2 and V3 were not notably higher than the original values based on data from deeper layers (Fig. 4), and *p* values for DIN and DIP for V1/V2/V3 did not increase (Table 5). The change of the data source had no apparent effect here.

#### The River Kymijoki

In KYMI, no evidence of an impact of the CF was found even at K2 (Table 5). The MF, in turn, was dominant between K2 and K3 (*p* < 0.05 for DIP for K2/K3). Furthermore, the DIN/DIP ratio collapsed from the highest observed level in the study at K1 into the offshore levels at K2 (Table 4 and Fig. 4). Apparently, marine water seems to dominate the chemical constituent of the water already in the primary recipient water basin.

The change of the data source to the surface layer data increased the DIN/DIP ratio at K2 to be ten times the ratio based on the original data, which suggested for a presence of waters high in N and poor in P in the surface layer at K2 (Fig. 4). The corresponding ratio at K3 located at the brink of outer archipelago was still three times the original ratio. In the original approach, there were no impacts of the CF according to both DIN and DIP, but the use of surface layer data suggested the predominance of the CF according to DIN landward from K3 (*p* < 0.05 for DIN for K1/K2/K3; Table 5). Waters in the surface layer at K2 and K3 were markedly of freshwater origin. As the original data provided the boundary area to locate between K1 and K2, the DIN content of the surface data suggested the area to locate between K3 and K4. The use of surface DIN data was the only way to show the land-based effect in the first place regardless of the massive impact of the River Kymijoki.

## Discussion

### Impact of analytical changes

Most of our station datasets commenced in the 1960s to the 1980s (Table 4) and thus were faced with a source of error stemming from technical changes in the laboratory analytics over the course of monitoring. The laboratory routine for DIN (except NH_4_) and DIP changed from manual determination to the autonomous samplers and analyzers. These issues have typically been addressed with proper instrument validation and inter-comparison procedures so as to maintain the representativeness of the monitoring dataset.

Natural variation obscures the assessment of the impact of this methodological variation on the long-term trophic trends. It emphasizes or compensates for the variation in a way that cannot be directly assessed; you cannot tell how much of the observed difference between, e.g., two annual representative values is due to natural variation and how much is due to the analytical fingerprint. Thus, we addressed this issue indirectly by comparing the variable to which the change in analytics had no impact ($\overline{a_{\mathrm{year}}}$) to the one having this impact ($\overline{a_{\mathrm{change}}}$).

The technical changes in the analytics seemed to play an insignificant role for the reliability of DIN and DIP estimates (Table 3), thus allowing us to draw reliable conclusions of the trophic development of the stations. The contrary result would have questioned the scientific foundations of this study. We do not know how widely the impact of technical changes in the analytics has been taken into account in the long-term environmental studies, but it is for certain that this has been rarely documented in the scientific literature. We suggest that the scientists working with long-term datasets would at least clarify the magnitude of this impact, just to be on the safe side.

The analytical outcome inherits all the errors due to in situ sampling as well, but as there was no information of the sampling details in the station metadata, the sources of error due to sampling were not within the scope of this study. This issue is dealt with extensively in the literature, e.g., Zhang (2007).

### Trophic development of the study cases

Even though most of our station datasets commenced in the 1960s to the 1980s, they still miss the early phases of anthropogenic eutrophication in the offshore GOF and the entire adverse development in the inner coastal area.

The eutrophication of the BS commenced in the early twentieth century and the most rigorous phase took place in the 1950s to the 1970s (Gustafsson et al. 2012; Kauppila et al. 2003). The eutrophication of the BS—being still in its accelerated phase at the time when the collection of our dataset started—was clearly seen in the offshore nutrient trends that were predominantly increasing; DIN in the 1980s and DIP in the 1990s (Fig. 3).

The eutrophication of the estuaries in the northern coast of the GOF started along the industrialization and intensification of agriculture in the late nineteenth century (Finni et al. 2001; Laakkonen and Lehtonen 1999). The point source loads into the waterways and the coastal area have decreased considerably since the worst situation in the middle of the twentieth century thanks to effective water pollution control measures. The scattered load has been more challenging to control, and clear decreases in the Finnish agricultural nutrient load into the BS have been detected as recently as at the turn of the millennium for phosphorus and even more recently for nitrogen (Ekholm et al. 2007; Rankinen et al. 2016). As we missed the main phase of advancing eutrophication in the coastal area, we tried to find some evidence of oligotrophication (Fig. 3). The only marked decreasing nutrient trends occurred in VANTAA due to reduction in the point-source load along the River Vantaanjoki and the estuary, the Vanhankaupunginlahti Bay (see below, Vahtera et al. 2010). In MUSTIO and KYMI, the lakes in the upper river basins have shown to buffer the changes in the catchment nutrient load from being reflected directly in the riverine nutrient concentrations (Huttunen et al. 2016).

### Extents of land-based and marine impacts

**Fig. 5:**
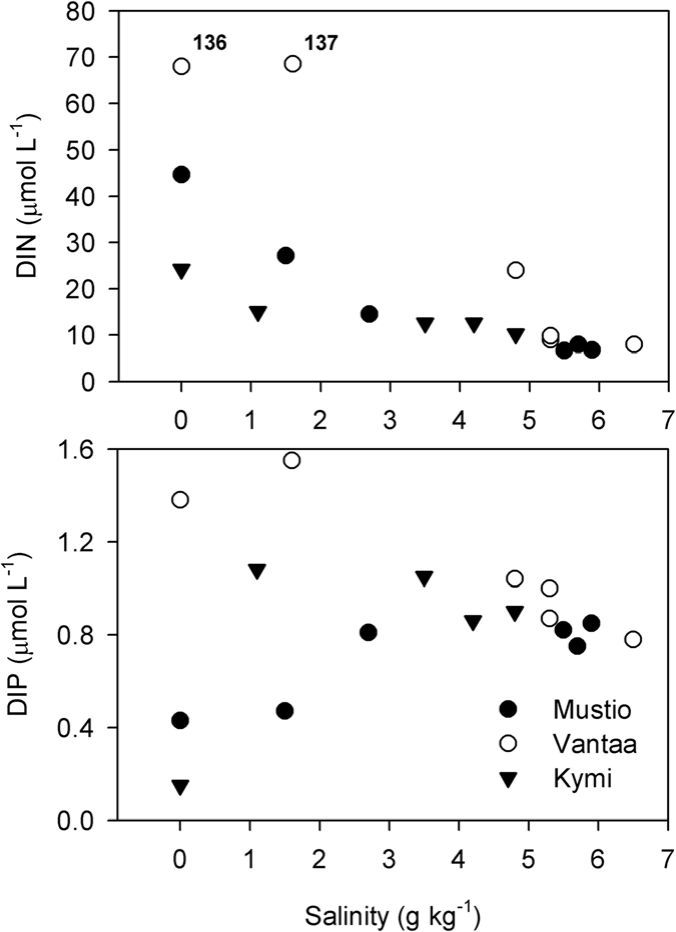
DIN and DIP as a function of salinity at the stations. The stations were named according to the regional initials (MUSTIO = M, VANTAA = V, and KYMI = K) and numbers according to the distance from the river mouth

Wintertime nutrient concentrations tend to behave conservatively along the coastal-offshore gradient (Officer 1979; Officer and Lynch 1981). The estuaries in the southern Finnish coast of the BS exhibited generally negative DIN-salinity gradients and positive DIP-salinity gradients (VANTAA as an exception) indicating that, on a large scale, the coastal area behaved consistently in terms of winter inorganic nutrients (Fig. 5). On a smaller scale, the variation in the land-based nutrient load and in the hydrodynamic/topographic conditions along the coastal-offshore gradient shaped the nutrient gradients and hence the extents of the CF and the MF on the coastal nutrient regime. This was especially seen in KYMI.

The CF was observed to be a predominant factor shaping the nutrient regime in the estuary and the inner coastal area along the coastal-offshore gradient but not beyond that; the MF was dominant in the outer coastal area. Kohonen and Mattila (2007) worked in the Turku, Åland, and Stockholm archipelagos in the BS that closely resemble the study area. They noted that the inner archipelago with poor water exchange rates was affected by the land-based nutrient load, while the outer archipelago with high water exchange rates was affected by nutrients from surrounding areas. This study supports this conclusion and further emphasizes the role of the estuary. The nutrient condition in the coastal-offshore gradient was decisively dependent on the characteristics of the estuary, the interaction of the riverine inflow with the waters of marine origin, and the areal topography determining the mixing and current patterns (Pritchard 1952).

#### The River Mustionjoki

The estuary of MUSTIO, the Pojo Bay, is kept stratified by episodic inflows of saltier water and only partial vertical mixing through the density gradient even at times of the inflows (Stipa 1996, 1999). The lack of continuous inflow of saltier water (Stipa 1999) suggests that the flow of the River Mustionjoki is too weak to trigger enhanced estuarine mixing, and the pronounced vertical salinity range at M2 (Table 2) suggests that the River is not able to break the salinity-driven stratification. The bay acts as a filtration mechanism producing a mix of fresh and already brackish waters. The narrow and shallow (6 m) Raasepori Sound that separates the bay from the GOF and the very shallow inner coastal area (< 3 m) maintain a two-layer system: an outflow rich in DIN in the surface and an inflow rich in DIP in the bottom. Consequently, the boundary area between the dominance of the CF and MF stretched seaward when we used DIN as diagnostics and landward when we used DIP. The determination of the boundary area, as we defined it, is problematic in an extremely scattered, land-dominated archipelago where the identification of the border between the estuarial and the truly marine systems depends on a viewpoint.

#### The River Vantaanjoki

Apart from the peak flow events, the flow of the River Vantaanjoki is too weak to be able to trigger enhanced estuarine mixing. Instead, a shallow (mean depth 4 m) and relatively open estuary leaves room for efficient wind-induced mixing also during mild and ice-free winter periods, which is apparent from a moderate vertical salinity range at V2 (Table 2). Even though the estuary is not stratified to such an extent that it could favor extensive river plumes, the riverine impact rich in nutrients is traceable throughout the inner coastal area. The inner part of the Helsinki archipelago shifts quite rapidly into the outer part (Fig. 1). Thus, the dominance of the CF shifts abruptly to that of the MF within reach of the basin-scale cyclonic current pattern.

#### The River Kymijoki

The high flow rate of the River Kymijoki triggers an enhanced estuarine mixing; a landward movement of deep water transports nutrients into the estuary (Pitkänen et al. 1986). Even though efficient mixing occurs in the boundary layer between the waters of riverine and marine origin (Pitkänen et al. 1986), the estuary is kept stratified with a sheer strength of the discharging river. The riverine inflow did not seemingly have any effect on the estuarine water characteristics with regard to nutrient content; the estuarine effect prevented any impact of the CF to be observed there. The impact was detected only with DIN in the surface layer. Still, it is safe to state that the substantial plume of the River Kymijoki dominates the chemical content of the surface layer in the estuary and has a marked influence on the surface layer all the way to the outer archipelago. Obviously, the monitoring network is too scarce to be able to properly tackle the intensive hydrodynamics.

### The study cases—typical coastal areas?

The southern seaboard of Finland is hydrologically segregated from the mainland catchments by the Salpausselkä ridge formations, and the area is characterized by about 20 small catchments discharging in the GOF (Database of the Finnish Environment Institute). All our cases were subject to a direct riverine nutrient impact. This is, however, not the case for a large part of the southern Finnish archipelago. The CF in a coastal-offshore gradient lacking a pronounced estuary hardly stretches as far away from the coast as it did in our cases, and hence, our approach probably produced an overestimate of the CF for a typical coastal strip. We could not test this hypothesis because the monitoring network in the southern Finnish archipelago is designed to tackle the direct riverine and/or anthropogenic impact. Therefore, the stations in the inner coastal areas devoid of the sources for riverine and/or point-source nutrient loads are scarce.

Bryhn et al. (2017) concluded that local-scale mitigation of eutrophication is only effective in those coastal water bodies which receive high nutrient inputs from the catchment as opposed to nutrients coming from the sea. Of our cases, local water management actions have made a large-scale difference in VANTAA. The Vanhankaupunginlahti Bay was subject to a pronounced municipal impact already in the nineteenth century (Laakkonen and Lehtonen 1999). In the 1960s, it was heavily polluted with municipal waste waters by the City of Helsinki (Varmo et al. 1989). The local municipal wastewater treatment plants were closed in the late 1980s and the wastewaters were directed to the central treatment facility discharging the effluents to the outer archipelago (Laakkonen and Lehtonen 1999). The water management actions made a difference in this case because much of the point-source load was relatively straightforward to be cut off and the reduced nutrient load was so substantial that the nutrient load from the eutrophic offshore waters could not compensate for the nutrient load reductions. To modify the message of Bryhn et al. (2017), local-scale mitigation of eutrophication in the coastal areas of the BS is successful and effective only when the land-based nutrient load is especially high, as the nutrient content of the BS is especially high for marine standards as well. This does not often take place in the BS; for instance, Bryhn et al. (2017) noted that in majority of Swedish coastal water bodies, nutrient input from surrounding marine waters was the most influential contributor among external nutrient fluxes.

### Implications for marine monitoring

The evaluation of the trophic regime of the sea has typically been based on the surface layer nitrogen and phosphorus content (e.g., HELCOM 2009; OSPAR 2009). This study suggests that this approach does not necessarily work for the estuaries. What makes the situation problematic is that estuaries are environments where the anthropogenic impact on the marine system is at its strongest (Borja et al. 2011; Fehér et al. 2012), and because of this, long estuarine datasets have often had a major impact on the messages provided by environmental state assessments.

The extents of the land-based and marine impacts on an estuary and the adjacent coastal area are considerably dependent on whether we choose to accept the surface layer data as part of the dataset or not. We have already shown this in our work, but to prove our point further, we compared the vertical DIP pattern of K2 to the one of the station UUS-22 locating off the Pernajanlahti Bay about 20 km to the west of K2. UUS-22 presents a typical southern Finnish coastal area of the BS under the impact of only small rivers. The deep DIP concentration level was much the same, 1.5 μmol L^−1^ at 29 m depth at UUS-22 and 1.7 μmol L^−1^ at 15 m depth at K2 (2000–2013 average, data not shown), whereas the surface DIP concentration level of UUS-22 was ten times the level of K2 (1.0 μmol L^−1^ at UUS-22 and 0.1 μmol L^−1^ at K2). The nutrient content of the estuarine surface layer reflects closely of that of the waters from the catchment especially in the wintertime, whereas the deeper waters are originated mainly from the offshore due to the density-driven estuarine circulation. The chemical content of the typical coastal water, however, does not resemble either of these cases but are a varying mixture of the two due to conservative behavior of the nutrients in the coastal-offshore gradient. This discrepancy becomes critical whenever the estuarine data are used in the state assessments.

We suggest that estuarine monitoring data should be managed as a separate case in the environmental assessments because the chemical environment of the estuarine water is often vertically biased. Estuarine datasets are invaluable when they describe the environmental responses to the long-term anthropogenic stress, but this value does not extend beyond the estuary.

### Implications for water management

The coastal areas of the BS are highly variable in terms of their hydrodynamic and topographic regimes determining their sensitivity to eutrophication, even in the GOF scale (Meeuwig et al. 2000; Pitkänen et al. 1993). The coastal-offshore gradient brings about another dimension into the dilemma; instead of referring to the coastal area in general, we are faced with how far off the coast we stand. This dimension sets challenges to marine monitoring and management. Here, we concentrate on the Finnish implementation of the European Marine Legislation.

The EU WFD (EU 2000) requires the EU Member States to use classification systems to describe the ecological status of their water bodies. The core of the system is the reference condition describing the pristine condition of the water body (REFCOND 2003), against which the ecological state is subsequently defined, and possible management measures implemented. Coastal-offshore gradients increase the already high natural variability within the Finnish coastal types of the EU WFD. Thus, the determination of the reference condition, and hence, the management quality standard (the line between the good and moderate states; REFCOND 2003) become less precise in various geographical parts of the type (Kauppila 2007). This introduces error in the socioeconomically essential determination between the good (= measures may not be needed) and less-than-good (= measures are obligatory) states.

In general, the estuaries are defined as belonging to transitional waters according to the EU WFD. The Finnish coastal water bodies were originally defined as belonging solely to coastal waters (Carletti and Heiskanen 2009; Kangas et al. 2003), even though some of them could be defined as belonging to transitional waters. Our results suggest that there is a clear justification for doing this re-definition in the future, a change from a coastal to a transitional water type. The Stockholm archipelago was already re-defined this way during the second management cycle in Swedish typology (Natursvårdsverket 2007).

If a number of Finnish coastal water bodies are re-defined this way, some large-scale implications will follow. The world’s estuarine–coastal ecosystems are continuously changing, but on top of that, the pace of change will likely accelerate along with the accelerating climate change (Cloern et al. 2016). Therefore, the minimum monitoring frequency indicated in the EU WFD is hardly adequate for describing the dynamic feature of transitional waters (Ferreira et al. 2007), especially thinking about the future, and the frequency for these water bodies should be increased in order to support the cost-effective marine management (Nygård et al. 2016). Second, transitional waters are considered to be naturally eutrophic in a comparison to coastal waters, so the reference conditions for these waters cannot not be the same (McLusky and Elliott 2007). The change in the reference condition would thus accompany the re-definition, having far-reaching consequences for their future classification.

The current use of transitional waters within the Baltic typologies is not coherent (Carletti and Heiskanen 2009; McLusky and Elliott 2007) and will not in the current state provide any consistence for the BS-scale state assessments. Should the introduction of transitional waters be chosen to be included in the Finnish surface water typology, special attention has to be drawn to the criteria for the selection of these water bodies and their reference condition, keeping in mind the difficulty of distinguishing between the effects of natural and anthropogenic stressors in these environments (Elliott and Quintino 2007).

## Conclusions

The scattered coastal area within the reach of riverine waters resembles variably the offshore area in terms of the nutrient content. We studied how far off the coast the coastal nutrient regime was predominantly dictated by the processes taken place in the catchment rather than in the sea. The catchment generally overrode the offshore waters as a primary nutrient source in the estuaries and throughout the inner coastal area, outside of which the basinwide current patterns controlled the nutrient condition. However, the hydrodynamic and topographic conditions along the coastal-offshore gradient, and especially those in the estuary, shaped the extent of the area under the domination of the catchment impact.

Monitoring of the marine environment and its resources is a key element for sustainable marine management. The value of marine monitoring data is an order of magnitude greater than the resources spent on collecting the data (Nygård et al. 2016), provided that the monitoring network properly represents the characteristics of the marine area. This study raises concerns of the value of the monitoring efforts targeted to estuaries to describe the environmental state of surrounding marine areas. Estuaries represent poorly the nutrient condition of the bulk of the coastal water formations, because estuaries do not reflect the processes and mechanisms occurring in the coastal area but rather outside it. There are two implications. First, the estuarine data should not be used to represent coastal waters but rather should be utilized separately in the environmental assessment process. Any generalization of the trophic condition of an estuary to represent areas farther off the coast should be done cautiously. Second, the estuarine water formations should be considered belonging to a transitional water typology instead of a coastal typology as it relates to European Marine Legislation.
